# Preparation, Characterization, Morphological and Particle Properties of Crystallized Palm-Based Methyl Ester Sulphonates (MES) Powder

**DOI:** 10.3390/molecules25112629

**Published:** 2020-06-05

**Authors:** Zulina Abd Maurad, Luqman Chuah Abdullah, Mohd Shamsul Anuar, Nor Nadiah Abdul Karim Shah, Zainab Idris

**Affiliations:** 1 Malaysian Palm Oil Board, 6, Persiaran Institusi, Bandar Baru Bangi 43000, Selangor, Malaysia; zainab@mpob.gov.my; 2 Department of Chemical and Environmental Engineering, Faculty of Engineering, Universiti Putra Malaysia, Serdang 43400, Selangor, Malaysia; 3 Department of Process and Food Technology, Faculty of Engineering, Universiti Putra Malaysia, Serdang 43400, Selangor, Malaysia; mshamsul@upm.edu.my (M.S.A.); nadiahkarim@upm.edu.my (N.N.A.K.S.)

**Keywords:** methyl ester sulphonates, purification, solvent crystallization, oleochemicals

## Abstract

Methyl ester sulphonates (MES) have been considered as an alternative green surfactant for the detergent market. Investigation on the purification of methyl ester sulphonates (MES) with various carbon chains of C_12_, C_14_, C_16_ and C_16–18_ derived from palm methyl ester is of great interest. These MES powders have been repeatedly crystallized with ethanol and the purity of MES has increased to a maximum of 99% active content and 96% crystallinity index without changing the structure. These crystallized MES with high active content have 1.0% to 2.3% moisture content and retained its di-salt content in the range of 5%. The crystallized MES C_16_ and C_16–18_ attained excellent flow characteristics. Morphology, structural and its crystallinity analyses showed that the crystals MES had good solubility properties, stable crystal structure (β polymorphic) and triclinic lateral structure when it is in high active content. The brittleness of MES crystals increased from a β’ to a β subcell. Crystal with high brittleness has the potential to ease production of powder, which leads to a reduction in the cost of production and improves efficiency.

## 1. Introduction

Methyl esters without any further modification are used as diesel substitute or biodiesel. However, due to the high content of palmitic acid and therefore higher pour point, methyl esters derived from palm oil or palm stearin are not suitable for the winter season. Palm olein needs to be used or palm methyl ester needs to be fractionated to obtain the more liquid methyl ester. With the rapid growth of the palm biodiesel where the production had increased 2.8 folds from 2016 to 2017, it will cause overcapacity of the saturated methyl ester [1]. Hence, there is a need to enhance the consumption of saturated methyl ester and one of the alternatives is to channel the saturated methyl ester as starting materials to produce methyl ester sulphonates (MES).

**Figure 1:**
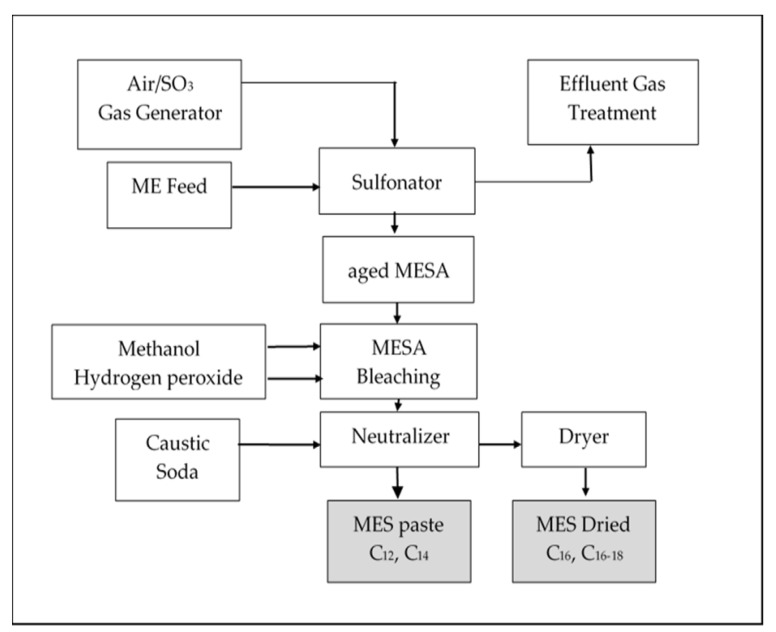
Block diagram for the methyl ester sulphonates (MES) process pilot plant at the Malaysian Palm Oil Board (MPOB).

The technology to produce MES is available worldwide; however, there are only a few producers of this surfactant in the world, such as Malaysian Palm Oil Board (MPOB), Chemithon Corporation (United States), Lion Corporation (Japan), KLK Oleomas (Malaysia), Stephan Company (United States), Wilmar International Ltd. (Indonesia), Guangzhou Keylink Chemical Co., Ltd. (China), Guangzhou Lonkey Industrial Co Ltd. (China) and Sinopec Jinling Petrochemical Co., Ltd. (China) [2,3]. The technology to produce palm-based MES involves sulphonation of a palm oil-based methyl ester with sulfur trioxide/air followed by digestion, bleaching and neutralizing to produce MES C_12_ and C_14_ and additional steps through the turbo tube drying and flaking system process to produce dry MES C_16_ and C_16–18_, as summarized in Figure 1 [4].

**Figure 2:**
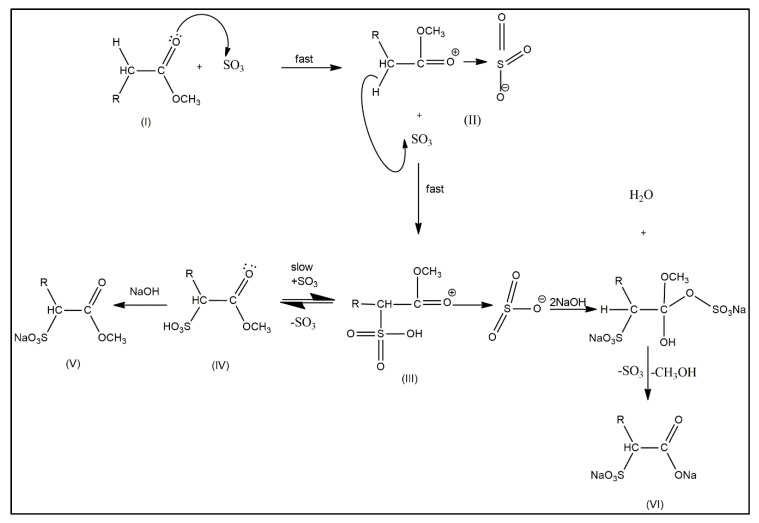
Reaction of methyl ester sulphonates (V) and the intermediates, such as di-salt (VI).

The mechanism for the formation of MES has been thoroughly studied and is believed to occur through several stages of the process, as in Figure 2 [5,6,7]. The first stage is when sulfur trioxide is reacted with saturated fatty methyl ester and rapidly produces mono-adduct (I). The mono-adduct attaches into an intermediate that activates the carbon at the position alpha to the carboxylic group; it will then be sulphonated with a second molecule of sulfur trioxide. Then, the di-adduct rearranges to form a dark color fatty methyl ester sulfonic acid (MESA) and release of one molecule of sulfur trioxide (II). The re-arrangement of the di-adduct is a limiting step and the process continues until all esters have been sulphonated (III). In the presence of an alkali, the fatty methyl ester sulfonic acid will form MES (V), but the di-adduct will hydrolyze into a di-salt (VI).

Research results have confirmed the superior properties of palm-based MES in terms of its detergency, biodegradation [8], synergy with soap [9] and tolerance to different water hardness in comparison to LAS. Furthermore, the cost of production of MES is comparatively lower than LAS [10]. These extensive research activities and testing have confirmed that MES and its cleaning products are environmental friendly [11]. The MES and formulated palm-based cleaning products are easily biodegraded [12].

A major application of MES was for the detergent segment; however, Grand View Research reported that the personal care segment was anticipated to emerge for over 34% of the MES market volume share by 2025 [13]. Application flexibility is largely based on the quality of the MES; in particular, the composition and physical form of the product. In Malaysia, MES C_16_ and C_16–18_ are currently commercially available in the market under the brand name of Mizulan and Palmfonate, with an active content of ~86% and generally used in detergents. MES C_12_ and C_14_ based on palm oil are not commercially available; however, MPOB has the capability to produce the middle cut with active contents of 68% and 83%, respectively, but contained a substantial amount of water, methanol, di-salt and other impurities. These impurities in MES C_12_ and C_14_ are irritants to the eye according to the *SkinEthicTM HCE* model [14]. Successful usage of MES as an active ingredient in personal care could be achieved if the MES C_12_ and C_14_ produced were non-irritant. The objective of this preliminary study was to investigate the feasibility of employing the crystallization process with ethanol as the solvent, to produce crystallized MES powder of various carbons with 97% to 99% active content and analyzing their properties for knowledge.

## 2. Results and Discussion

### 2.1. Crystallized MES Powder

**Figure 3:**
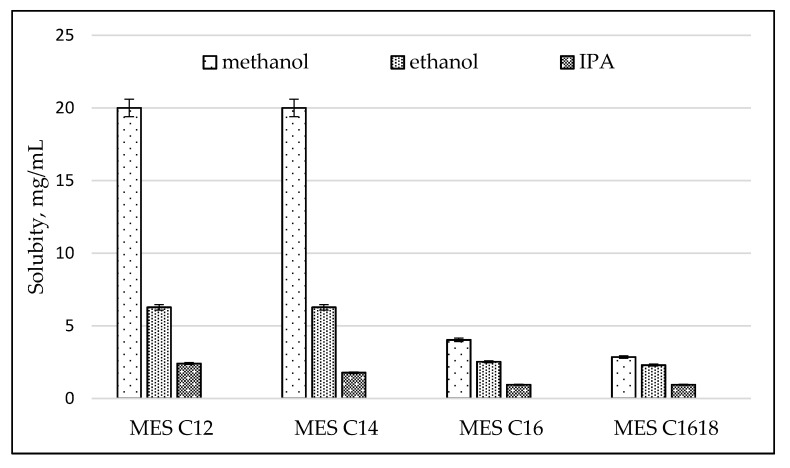
Solubility of MES in solvents.

Crystallized MES powder of various carbon chain lengths (C_12_, C_14_, C_16_ and C_16–18_) with 97% to 99% active content had been obtained through grinding, crystallization and drying processes. These processes were to eliminate moisture content and increase active content but retain the di-salt content. Four solvents, such as methanol, ethanol, isopropyl alcohol (IPA) and hexane, were selected to determine the solubility of MES, as in Figure 3. Ethanol was the second-highest solubility polar solvent having 6.3-2.5 mg/mL solubility with MES. MES C_12_ has the highest solvent solubility rates compared to other carbon chain lengths. The shorter the carbon chain of MES, the higher the solvent solubility rate. If the carbon chain of MES is large, the critical micelle concentration (CMC) and solubility should decrease [15,16]. Methanol shall not be used as solvent in the purification of MES as it can increase di-salt content (undesirable) and is extremely toxic [17]. Ethanol is the most suitable solvent for the crystallization process of MES. In addition, ethanol can be made from a renewable energy fuel from sugar crops, starches or cellulosic biomass [18]. In this study, MES was not dissolved in hexane. The overall cost-benefit using co-solvent (ethanol and water) with low toxicity solvent in repeated crystallization of MES is high. The solvent crystallization process can increase product stability, which can have a positive influence in reducing manufacturing costs. However, the cost of disposal of the used solvent must be taken into consideration.

The dried crystallized MES powder produced from repeated crystallization from ethanol has an active content of MES C_12_: 98.96%, MES C_14_: 97.76%, MES C_16_: 99.32% and MES C_16–18_: 99.18%. The active content of MES C_14_, C_16_ and C_16–18_ has an increment of 15% to 19% while MES C_12_ has the highest active increment of 45%. Moisture content of MES has been dried to 1.0% to 2.3%, while di-salt content was retained less than 5%, which is desirable. A higher di-salt content leads to a lower surface activity of MES and lower hardness, as di-salt’s Krafft point is higher than that of MES [19]. Crystallization can also be seen as a technique to obtain solid products, as MES C_12_ and C_14_ after repeated crystallization becomes solid powder from a paste. Other specifications of MES are shown in Table 1.

### 2.2. Structural Conformation of Crystallized MES Powder

**Figure 4:**
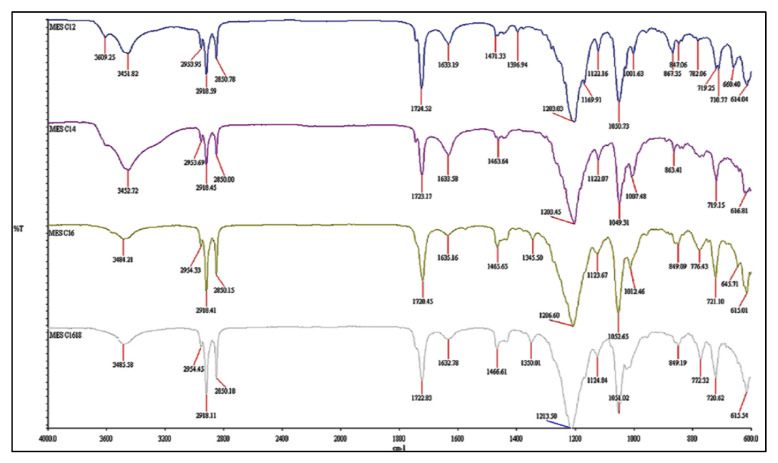
Infrared of MES before crystallization.

**Figure 5:**
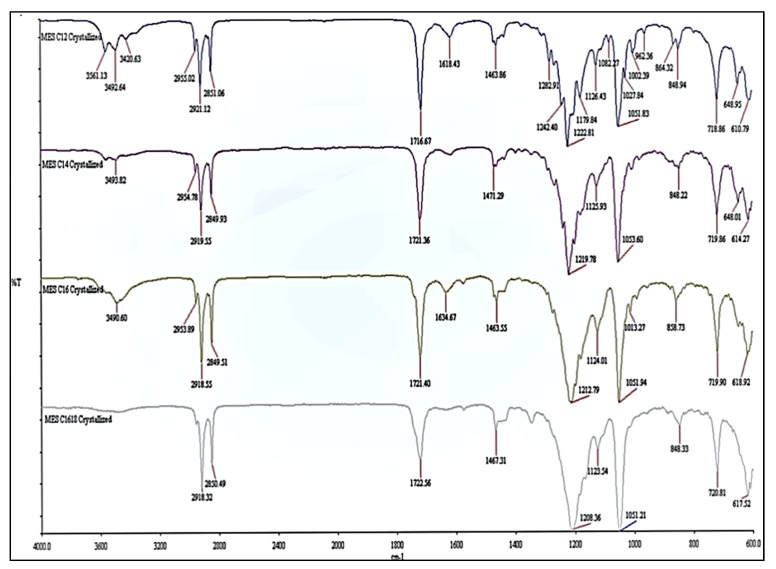
Infrared of MES after repeated crystallization.

The infrared spectrum of MES before and after repeated crystallization are illustrated in Figure 4; Figure 5, respectively. Both infrareds have the same band pattern except for MES after repeated crystallization, which has lower intensity at 3611–3420 cm^−1^ (-OH stretching) and 1640–1600 cm^−1^ (H–O–H bending region) compared to infrared from MES before crystallization. This is due to lower moisture content in high active content MES after repeated crystallization and is in agreement with the study conducted by Hongping et al. [20]. The typical peaks of the methyl group (C–H) at 2958–2849 cm^−1^ and carbonyl group (C=O) at 1724–1716 cm^−1^ were observed in both infrared. The presence of the sulfonate group (S=O) stretching vibration at the strong peak 1222–1051 cm^−1^ and (S–O) stretching at peak 857–719 cm^−1^ indicates that both compounds are methyl ester sulfonate [21,22]. The crystallization does not affect the structure of MES, but instead increased the active content and reduced the moisture content.

The composition of MES before and after repeated crystallization was similar and confirmed by ^1^H and ^13^C NMR analysis (Table 2 and Table 3). The MES was dissolved in deuterium water (D_2_O) and processed at 60 °C. A proton attached to the carbon atom (–CH–), which, bearing the sulfonate group, appears at peak 3.87–3.89 ppm (s, H). The peak for the hydrogen attaching to the carbon atom bearing an ester group (–COO) appears at 3.71–3.75 ppm (t, 3H). The hydrocarbon chain is observed at the peak between 0.76 and 0.80 ppm (terminal CH_3_–) and 1.90 to 2.00 ppm (CH_2_– linked with –CH). The difference between various MES chain lengths is at peak 1.16 to 1.21 ppm (CH_2_) _n_- with MES C_12_ (d, 16H), MES C_14_ (d, 20H), MES C_16_ (d, 24H) and MES C_16–18_ (d, 28H).

The ^13^C-NMR spectra for all MES before and after repeated crystallization indicated that the carbon for the terminal methyl (CH_3_) appears at peak 13.86 to 13.95 ppm. The carbon directly attached to the sulfonate group (–SO_3_Na–CH–) is observed at peak 66.02 to 66.20 ppm. The signal for carbon directly attached to the ester group (–OCH_3_–) is observed at peak 52.96 to 53.06 ppm and the carbon (C=O) appears at peak 170.52 to 170.59 ppm. Both FTIR and NMR results are in good agreement with the results obtained from the literature and prove that all are MES products [23].

### 2.3. Properties of Crystallized MES Powder 

#### 2.3.1. Surface Morphology

**Figure 6:**
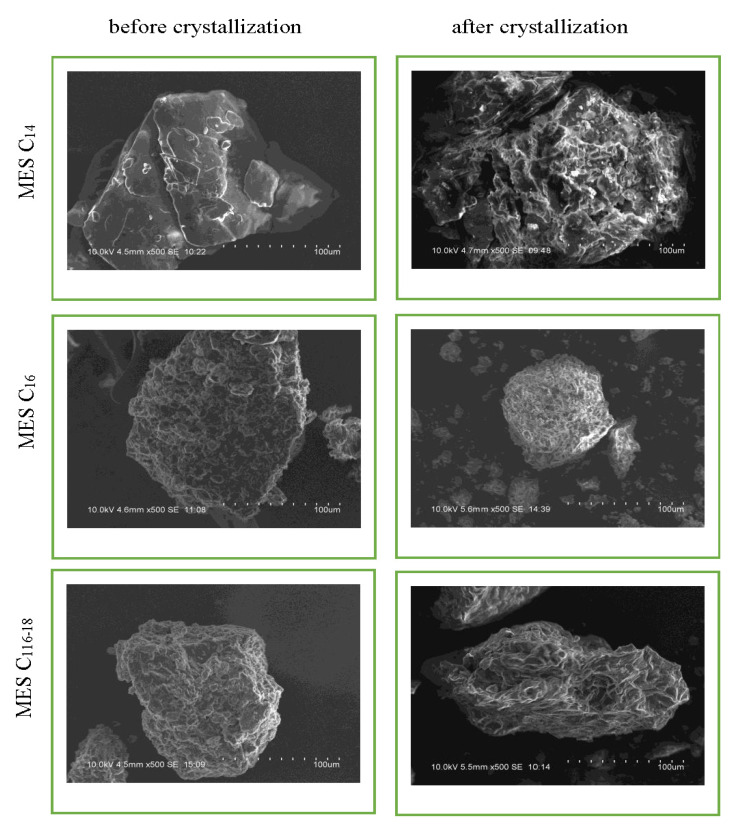
Surface morphology of the MES before and after repeated crystallization.

The surface morphology of the MES before and after repeated crystallization was examined by scanning electron microscopy analysis (SEM), as shown in Figure 6. Both MES before and after repeated crystallization have an elongated spherical particle structure in clusters or in an agglomerated form, which is in agreement with a study conducted by Babu et al. [24]. Also, the particles have irregular shapes and sizes. The elemental analysis values found from energy dispersive x-ray spectroscopy (EDX) had confirmed the oxygen, sodium and sulfur contents in the MES, and values are tabulated in Table 4. No other peak for any other element can be found in the spectrums, which supports that the surfactant is MES. The surface morphology of MES C_12_ cannot be compared from before and after repeated crystallization as, initially, MES C_12_ is in paste form.

#### 2.3.2. Particle Size (Dynamic Light Scattering)

Table 5 shows that the median diameter of particle MES increased after crystallization. With the increase in MES surfactant active content, the particle size increases simultaneously [25]. The increase of mean size diameter results from the aggregation of the molecules. Intergrowth of aggregates formed by particle collisions, through a crystallization process that forms an agglomerative bond [26].

#### 2.3.3. Flow Behavior

**Figure 7:**
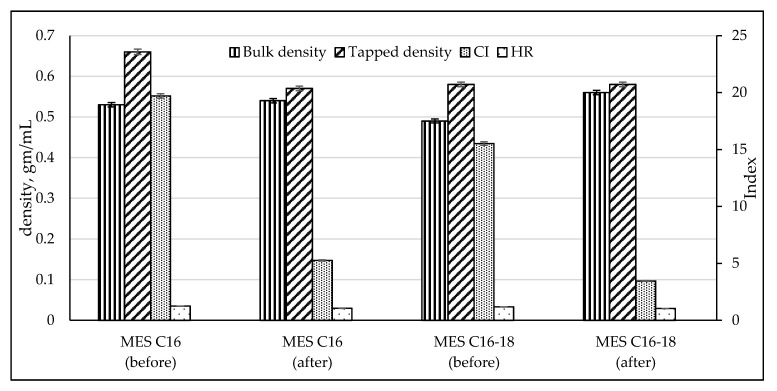
The bulk and tapped density of palm stearin-based MES (C_16_ and C_16–18_) before and after repeated recrystallization.

The bulk and tapped density of palm stearin-based MES (C_16_ and C_16–18_) before and after repeated recrystallization were evaluated in order to determine the flowability of the powder through the Carr Compressibility Index (CI) and Hausner Ratio (HR) (Figure 7). Powder MES of C_16_ and C_16–18_ before crystallization had fair and good flow characteristics, respectively, due to the existence of larger interparticle interactions; thus, a greater difference between bulk and tapped densities is observed. The HR is also affected by normal and tangential not-roundness. As the not-roundness of the cross-sectional shape increases, so too does the HR increase [27]. After repeated recrystallization, MES of C_16_ and C_16–18_ with high active content attained excellent flow characteristics. Therefore, interparticle interactions are generally less significant, for which the bulk and tapped densities have been relatively close in magnitude. In addition, the particles of MES after repeated crystallization is in pure homogeneity form.

#### 2.3.4. Crystallinity

**Figure 8:**
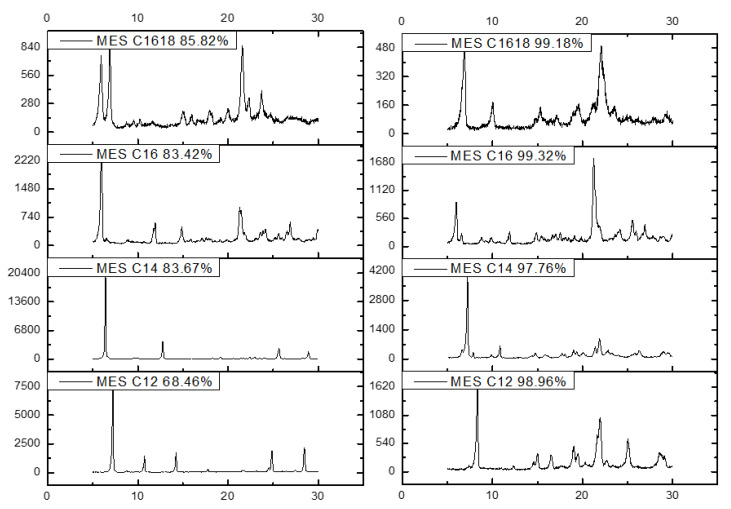
X-ray diffraction (XRD)-histograms of MES C_12_, C_14_, C_16_ and C_16–18_ before (left) and after crystallization (right).

The detail about the crystal of MES has been evaluated using x-ray diffraction (XRD). The histograms in Figure 8 show the small angle scattering (SAXS) at 1° < 2θ < 12° and wide-angle x-ray scattering (WAXS) at 12° < 2θ < 35° of MES C_12_, C_14_, C_16_ and C_16–18_ before (low active content) and after repeated crystallization (high active content). The SAXS pattern of MES gives information about the lamellae and crystal thickness while WAXS gives information on polymorphism of the MES. 

A study by Larsson [28] states that the primary forms of polymorphic are α, β’ and β. From the histograms, the MES C_12_, C_14_, C_16_ and C_16–18_ after repeated crystallization (high active content) and MES C_16–18_ before crystallization has a β polymorphic form, which is the most stable arrangement and will result in a coarse and grainy texture [29]. This is due to the peak that appears at positions d = 4.6, 3.8 and 3.7 Å. The Bragg peaks define these subcells within the crystal lattice of a triclinic [30]. The MES C_12_ and C_14_, before (low active content), do not have any peak position for α, β’ and β but MES C_16_ has peaks appearing at positions d = 4.2 and 3.8 Å, which confirms the β’ polymorphic (orthorhombic crystal lattice) form according to the XRD AOCS method [31].

The crystalline structure of MES was transformed from metastable crystal (α subcell) to a mixture of anhydrous crystals (β subcell) and dihydrate crystals (β’ subcell) owing to melt-mediated crystallization. The brittleness of MES crystals increased from an α to a β’ to a β subcell. Transformation into more brittle crystals will lead to a decreased yield value of MES [32]. Crystals with low brittleness are disadvantageous during manufacturing processes because enormous amounts of energy are required to convert them into powder.

**Figure 9:**
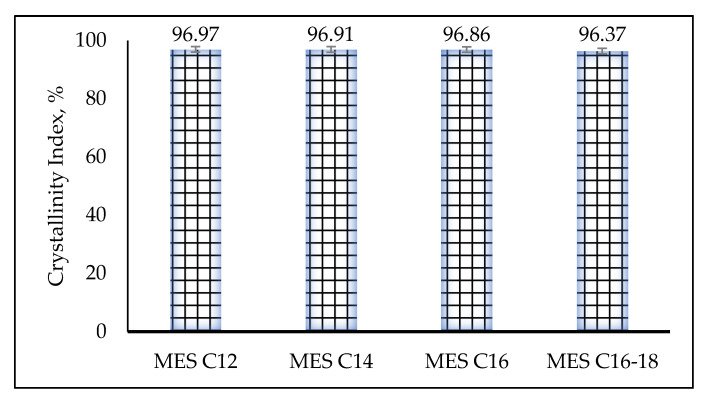
Crystallinity Index (CrI) of MES after repeated crystallization.

The crystallized MES (C_12_, C_14_, C_16_ and C_16–18_) has a crystallinity index more than 96% (Figure 9) based on the Segal equation, as stated in the equation [33]: Crystallinity Index, $\mathrm{CrI}\;\left(\%\right)=\frac{I_{002}-I_{am}}{I_{002}}X\;100$. The Segal method is based on two parts of crystalline and amorphous. The amount of crystalline material is implied by the height of the highest diffraction peak (*I_002_*), and the amount of amorphous material is implied by the height of the minimum intensity between the major peaks (*I_am_*). The CI is the difference between these two intensities, divided by the intensity of the highest peak (*I_002_*).

#### 2.3.5. Solubility

**Figure 10:**
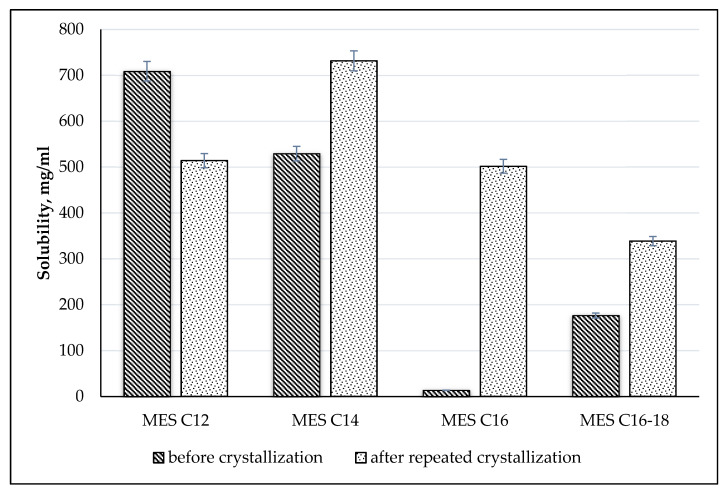
Solubility of MES with different active content in water.

Good water solubility properties at ambient temperature make the product easy to incorporate in liquid formulations. The solubility of MES before crystallization and after repeated crystallization has been determined (Figure 10). The higher the purity of MES (C_14_, C_16_ and C_16–18_), the better the solubility except for MES C_12_ because impurities generally lead to reduced CMCs, thus reduce the solubility [34]. MES C_12_ before crystallization is already in liquid form and has a hydrogen bond and is, therefore, easier to dissolve. The pure MES C_14_ has higher solubility compared to MES C_16_ and MES C_16–18_. The solubility decreases with an increase in molecular weight [35].

#### 2.3.6. Melting Point

A melting point is a useful indicator of purity as there is a general lowering and broadening of the melting range as impurities increase. Table 6 indicates the melting point of MES with different purities according to active content. The higher the active content of MES of each chain length, the greater the melting point increase; thus, the impurities have been eliminated. The melting point of alkyl α-sulfopalmitates (C_14_) and stearates (C_16_) has been reported by Weil et al. with values of 180.9–182.8 and 179.8–180.0 °C, respectively, but their preparation in a state of purity has not been adequately described [36].

## 3. Materials and Methods

### 3.1. Materials

MES produced from MPOB usually has an active content of 83% to 86% for MES C_14_, C_16_ and C_16–18_ and 68% for lower chain MES C_12_. It has very low color (<25 Klett color) and low di-salt content, (<5%). Other specifications as listed in Table 7.

### 3.2. Preparation of Crystallized MES Powder

MES of various carbon chain lengths (C_12_, C_14_, C_16_ and C_16–18_) were highly purified by repeated recrystallization from ethanol. MES C_12_ and C_14_ were in semi-liquid and paste forms, respectively. While MES C_16_ and C_16–18_ that are in flake form will need to be grounded until a particle size of at least of 700 to 750 µm is achieved using a high shear knife mixer. No preheating was needed to prevent the sample from evaporating. MES was then mixed with hot ethanol/ distilled water mixtures (95/5 by volume) and stirred using a magnetic bar with 1000 to 1050 rpm for 20 to 60 min depending on the physical form of MES and the solubility properties. The total amount of solvent used with the MES to solvent ratio (wt/wt%) were 1:1, 1:2 and 1:3 depending on the concentration of the mixture. The mixture of MES and solvent were left to cool down and crystallize in a glass refrigerator with a temperature of 4.5 to 5 °C without any mechanical induced convective airflow. The crystallization time was set to 24 h. After 24 h of crystallization, the crystals were emptied onto a bush funnel and filter paper with a 6 µm pore size and connected to a jet water aspirator to perform vacuum filtration. The filtration process to segregate the MES crystals from the mother liquor took 240 min. The semi-dry crystal particles MES were recrystallized again for another two or three cycles to obtain highly purified MES. After the repeated recrystallization, the semi-dry crystal particles MES were scattered on plastic trays and subjected to mechanically induced ventilation for 24 h. This drying process was carried out under normal room temperature at 27 °C. The dried MES crystal particles were then stored in the presence of silica gel as a drying agent. The yield of repeated crystallization for MES C_14_, C_16_ and C_16–18_ was 96% and a lower yield for MES C_12_ with 85%. All experiments and analyses were conducted in duplicates in order to ensure reproducibility.

### 3.3. Analyses

#### 3.3.1. MES Specifications

The method for specifications of MES was described by Battaglini et al. [37]. The di-salt content was determined from two titrations (methylene blue and phenol red titration) and a result from the free oil determination. The measurement for color was determined by Klett–Summerson colorimeter (Bel-Art Products, Scienceware, USA). All analyses were performed in duplicate with data reported representing average values.

#### 3.3.2. Active Content 

The active component or purity of the surfactant in detergent was determined by titration procedure (anionic) according to The Chemithon Analytical Method 1101.2 [38] based on ASTM D3049 [39]. The analysis was performed in triplicate with data reported representing average values. 

#### 3.3.3. Moisture Content

The moisture content of MES was determined using the Mettler Toledo, C30 Karl Fischer Compact Titrator (Switzerland) and the method specified by Scholz [40] according to AOCS Ca 2e-84.

#### 3.3.4. Fourier Transform—Infrared Analysis

The Fourier Transform—Infrared spectroscopy (FTIR) analysis was carried out to determine the functional group(s) present in the MES compound according to Nikolic [41]. This Spectrum 100 equipped with attenuated total reflectance unit was from Perkin Elmer (USA). The spectral range in wave-numbers for the spectrophotometer was set at 4000 to 600 cm^-1^, and the final output presentation was in percent transmittance.

#### 3.3.5. Nuclear Magnetic Resonance Analysis (NMR)

The structure of MES was identified by ^1^H and ^13^C NMR analyses with deuterium water as the solvent using a JOEL RESONANCE, model EC2 600R, 600 MHz (Japan).

#### 3.3.6. Scanning Electron Microscopy Analysis

The Scanning Electron Microscopy (SEM) analysis was carried out to observe the morphology of the MES crystals formed using Hitachi S-3400N (Japan) coupled with energy dispersive x-ray spectroscopy (EDX), model Bruker X Flash 6I10 (US). The Quantax System, ESPRIT software was used for data acquisition and analysis. 

#### 3.3.7. Particle Size Distribution Analysis

The Particle Size Distribution (PSD) of powder MES was measured in ethanol by using Malvern Instruments Mastersizer 2000 and the Hydro 2000S particle size analyzer (United Kingdom) at room temperature, 27 °C. Each sample was repeated in duplicate to ensure good repeatability. The PSD was characterized by the median diameter. 

#### 3.3.8. Flow behavior

The Carr Compressibility Index (CI) and Hausner Ratio (HR) are two measures that can be used to predict the propensity of a given powder sample to be compressed, and which are understood to reflect the importance of interparticle interactions. The CI and HI are calculated using the following relations:

(1) $$\mathrm{Carr}\;\mathrm{Compressibility}\;\mathrm{Index}\;(\mathrm{CI})=\frac{\mathrm{tapped}\;\mathrm{density}\;-\;\mathrm{bulk}\;\mathrm{density}}{\mathrm{tapped}\;\mathrm{density}}\;\times \;100$$

(2) $$\mathrm{Hausner}\;\mathrm{Ratio}\;(\mathrm{HR})=\frac{\mathrm{tapped}\;\mathrm{density}}{\mathrm{bulk}\;\mathrm{density}}$$

A CI of <10 or HR of <1.11 is considered ‘excellent’ flow whereas CI  >  38 or HR  >  1.60 is considered ‘very very poor’ flow. There are intermediate scales for CI between 11 and 15 or HR between 1.12 and 1.18 is considered ‘good’ flow, CI between 16 and 20 or HR between 1.19 and 1.25 is considered ‘fair’ flow, CI between 21 and 25 or HR between 1.26 and 1.34 is considered passable flow, CI between 26 and 31 or HR between 1.35 and 1.45 is considered ‘poor’ flow, and CI between 32 and 37 or HR between 1.46 and 1.59 is considered ‘very poor’ flow [42,43].

#### 3.3.9. X-ray Diffraction

The powder X-ray diffraction (XRD) measurement was carried out using XRD 6000 Shimadzu (Japan) equipped with basic process software for data acquisition and analysis. About a 200–500 mg sample was ground and mounted on an alumina sample holder. Data were acquired using a Cu monochromatized radiation source operated at 30 kV and 30 mA. The intensity data were recorded by a continuous scan in the diffraction angle 2θ from 5° to 30° with a step size of 0.02°. 

#### 3.3.10. Melting Point

The melting point of MES was determined according to a capillary tube method using Electrothermal 9100 (Essex, UK). 

## 4. Conclusions

Purification of MES powder has been conducted through a crystallization process with ethanol as the solvent. The process successfully achieved more than 97% active content instead of 86%, 1.0% to 2.3% moisture content and retained its di-salt content in the range of 5%. Through crystallization also, MES C_12_ and C_14_ were converted to solid powder. The composition of MES before and after repeated crystallization were similar and confirmed by infrared spectrum, ^1^H and ^13^C NMR analysis. Crystallized MES powder has an elongated spherical particle structure in clusters or in an agglomerated form. With the increase in MES surfactant active content, the particle size increases simultaneously. Crystallized MES C_16_ and C_16–18_ with high active content attained excellent flow characteristics. Crystallized MES C_12_, C_14_, C_16_ and C_16–18_ with high active content have a β polymorphic form and triclinic lateral structure, which is the most stable arrangement, and have a crystallinity index more than 96%. The brittleness of MES crystals increased when at the β subcell form. Therefore, the production of powder will require high brittleness of MES to reduce the cost. The higher the purity of MES, the better the solubility. The solubility decreases with an increase in molecular weight. The crystallized MES C_12_, C_14_, C_16_ and C_16–18_ with high active content have melting points of 203, 217, 197 and 229 °C, respectively. The crystallization of MES is a great process to produce high active MES for product specification.

## 5. Patents

There is patent filed number PI2019005429, resulting from the work reported in this manuscript.

## Figures and Tables

**Table 1:** Specifications of high active content crystallized MES powder.

Product/Parameter	MES C_12_	MES C_14_	MES C_16_	MES C_16–18_
Molecular weight	316.47	344.07	372.46	382.71
Active (%)	98.96 ± 0.04	97.76 ± 0.02	99.32 ± 0.04	99.18 ± 0.06
Di-salt/active (%)	0.42 ± 0.03	4.46 ± 0.03	4.76 ± 0.04	4.72 ± 0.01
5% Klett	11.95 ± 0.23	2.03 ± 0.45	18.08 ± 0.12	25.20 ± 0.34
pH (10%)	5.42 ± 0.02	3.89 ± 0.04	5.26 ± 0.01	5.44 ± 0.01
Moisture (%)	2.32 ± 0.12	1.03 ± 0.43	1.74 ± 0.31	2.20 ± 0.09
Appearance	-----------------------Powder crystals--------------------			

**Table 2:** NMR spectra data of MES before and after repeated crystallization.

MES	Acyl Chain Length	Assignment (δ ppm)				
		Terminal CH_3_–	–(CH_2_) _n_–	CH_2_– Linked with –CH–	–OCH_3_	S–CH–
		Before Crystallization				
C_12_	12	0.74–0.76	1.17	1.92–1.97	3.71	3.87
		(t, 3H)	(d, 16H)	(d, 2H)	(t, 3H)	(s, H)
C_14_	14	0.73–0.76	1.16	1.90–1.95	3.71	3.87
		(t, 3H)	(d, 16H)	(d, 2H)	(t, 3H)	(s, H)
C_16_	16	0.73–0.76	1.17	1.91–1.96	3.71	3.87
		(t, 3H)	(d, 16H)	(d, 2H)	(t, 3H)	(s, H)
C_16–18_	16–18	0.73–0.77	1.18	1.92–1.97	3.71	3.87
		(t, 3H)	(d, 16H)	(d, 2H)	(t, 3H)	(s, H)
		**After Repeated Crystallization**				
C_12_	12	0.78–0.80	1.21	2.00	3.75	3.89
		(t, 3H)	(d, 16H)	(d, 2H)	(t, 3H)	(s, H)
C_14_	14	0.74–0.76	1.17	1.95	3.74	3.89
		(t, 3H)	(d, 16H)	(d, 2H)	(t, 3H)	(s, H)
C_16_	16	0.74–0.76	1.17	1.99	3.73	3.89
		(t, 3H)	(d, 16H)	(d, 2H)	(t, 3H)	(s, H)
C_16–18_	16–18	0.74–0.76	1.17	2.00	3.74	3.89
		(t, 3H)	(d, 16H)	(d, 2H)	(t, 3H)	(s, H)

**Table 3:** Nuclear magnetic resonance analysis (NMR) spectra data of MES before and after repeated crystallization.

MES	Assignment (δ ppm)					
	Terminal CH_3–_	Adjacent (CH_2_)–	(CH_2_)_n_–	–OCH_3_	–CH–	C=O
	Before Crystallization					
C_12_	13.86	22.68	27.39–32.06	53.04	66.09	170.54
C_14_	13.86	22.71	27.43–32.10	53.00	66.20	170.52
C_16_	13.93	22.74	27.47–32.13	52.97	66.04	170.52
C_16–18_	13.93	22.72	27.49–32.16	52.96	66.02	170.52
	**After Repeated Crystallization**					
C_12_	13.88	22.71	27.43–32.09	53.06	66.14	170.59
C_14_	13.89	22.74	27.48–32.13	53.03	66.12	170.58
C_16_	13.89	22.76	27.52–32.15	52.99	66.07	170.57
C_16–18_	13.95	22.79	27.56–32.17	52.98	66.06	170.56

**Table 4:** Elemental analyses of MES.

MES	Elemental Analyses, %					
	Before Crystallization			After Crystallization		
	O	Na	S	O	Na	S
C_14_	16.44 ± 4.83	11.95 ± 2.67	14.64 ± 0.16	18.76 ± 4.89	15.22 ± 4.75	17.05 ± 1.65
C_16_	16.72 ± 3.99	12.15 ± 1.82	15.69 ± 1.32	13.97 ± 13.62	12.18 ± 10.92	17.23 ± 1.12
C_16–18_	19.20 ± 5.76	13.62 ± 3.70	14.65 ± 0.43	10.98 ± 8.13	8.84 ± 4.97	13.50 ± 0.87

**Table 5:** Particle size of MES surfactant.

Parameter	Mean Size Diameter (µm)			
	MES C_12_	MES C_14_	MES C_16_	MES C_16–18_
***MES before crystallization***	liquid	72.59 ± 0.44	9.99 ± 0.25	404.87 ± 1.16
***MES after crystallization***	5.45 ± 1.11	73.74 ± 0.11	12.52 ± 0.35	447.18 ± 1.25

**Table 6:** Melting point of MES.

Parameter	Melting Point Capillary Tube, °C			
	MES C_12_	MES C_14_	MES C_16_	MES C_16–18_
Before crystallization	131.0 ± 0.4	116.3 ± 0.2	186.5 ± 0.1	209.2 ± 0.3
After repeated crystallization	203.1 ± 0.2	217.8 ± 0.4	197.8 ± 0.0	229.5 ± 0.4

**Table 7:** Technical specifications of MES with different carbon chain lengths produced from MPOB’s MES plant.

Product/Parameter	MES C_12_	MES C_14_	MES C_16_	MES C_16–18_
Molecular weight	316.47	344.07	372.46	382.71
Active (%)	68.46 ± 0.07	83.67 ± 0.05	83.42 ± 0.06	85.82 ± 0.09
Di-salt/active (%)	1.03 ± 0.05	4.18 ± 0.07	4.30 ± 0.10	4.02 ± 0.07
5% Klett colour	7.14 ± 0.11	20.23 ± 0.15	21.89 ± 0.09	24.11 ± 0.12
pH (10%)	4.88 ± 0.03	3.29 ± 0.02	5.38 ± 0.03	5.52 ± 0.02
Moisture (%)	21.95 ± 0.24	14.03 ± 0.25	9.20 ± 0.19	8.92 ± 0.22
Appearance	semi-liquid	paste	flakes	flakes
